# Problematic Internet Use in adolescents: New psychometric evidence for the Spanish short form of the Compulsive Internet Use Scale

**DOI:** 10.1002/brb3.3133

**Published:** 2023-08-02

**Authors:** Julia Pérez‐Sáenz, Javier Ortuño‐Sierra, Alicia Pérez‐Albéniz, Oliver Mason, Eduardo Fonseca‐Pedrero

**Affiliations:** ^1^ Educational Sciences Department University of La Rioja Logroño Spain; ^2^ Programa Riojano de Investigación en Salud Mental (PRISMA) Logroño Spain; ^3^ School of Psychology University of Surrey Guildford UK

**Keywords:** adolescence, CIUS‐S, measurement invariance, problematic internet use, psychometric properties

## Abstract

**Objectives:**

Problematic Internet Use (PIU) has become a worldwide problem in recent years. Among screening instruments for PIU, the Compulsive Internet Use Scale (CIUS) is perhaps the most widely used. Psychometric properties of the full CIUS are not convincing, however, and the short form (CIUS‐S) has shown promising results, albeit limited to the English version, with little evidence in Spanish. Therefore, the aim of the present work was to study the psychometric properties of the CIUS‐S scores in a large sample of Spanish adolescents.

**Method:**

The sample consisted of 1790 participants, 816 male (45.6%), 961 female (53.7%), and 13 other (0.7%) . Mean age was 15.70 years (*SD* = 1.26).

**Results:**

The five‐items one‐dimensional model displayed appropriate goodness‐of‐fit indices. Strong measurement invariance model across age and partial across gender was found. Furthermore, the CIUS‐S was positively associated with several indicators of poor well‐being and mental health, and negatively associated with prosocial behavior, self‐esteem, and feeling of belonging.

**Conclusion:**

Overall, the CIUS‐S scores appear reliable and valid in its Spanish version for adolescent populations, supporting its aim to detect and prevent a problem that has become a major worldwide issue in the last years.

## Abstract 2

Short form of the Compulsive Internet Use Scale (CIUS) among adolescents.

## 1 INTRODUCTION

Several relevant constructs like identity, self‐concept, self‐esteem, and lifestyle consolidate during adolescence, a period of life with particular physical, psychological, and social changes (Goldbeck et al., 2007; Liu et al., 2022). Thus, adolescence is considered a stage of potential vulnerability for different psychological problems, including Problematic Internet Use (PIU) (Rial et al., 2015). Not surprisingly, prevalence rates of psychological problems during this period range between 10% and 20% (de Vries et al., 2018). Thus, adolescence is a crucial developmental stage during which it is important to implement prevention and treatment of psychological problems (Aritio‐Solana et al., 2022; Fonseca‐Pedrero et al., 2021).

PIU is defined as a generalized and compulsive use of the Internet associated with a loss of control and negative consequences for the individual (Caplan, 2002, 2010). It occurs when individuals remain connected for long periods of time, mostly for activities not related to work or study, causing difficulties at school, family, and social relationships (Sarmiento et al., 2021). Numerous studies have examined the psychopathological comorbidity between different mental health problems and PIU (Anderson et al., 2017; Shapira et al., 2003; Tsitsika et al., 2014; Werling & Grünblatt, 2022). Depression, anxiety, attention deficit hyperactivity disorder, and substance use disorders, among others, have shown a relationship with PIU (Ioannidis et al., 2018; Rial et al., 2014; Sussman et al., 2018). Some studies also suggest that PUI may be associated with increased rates of suicidal behaviors and/or self‐harm (Herruzo et al., 2023) and psychotic experiences (Lee et al., 2019). PIU has received increasing research and clinical attention, but it has not yet been recognized by diagnostic classification systems. Indeed, it is not clear whether it should be considered as a mental disorder or it just reflects other underlying mental health disorders (Chamberlain et al., 2018; Vink et al., 2016). The 11th revision of the International Classification of Diseases (ICD‐11; World Health Organization, 2019) includes two behaviors (gambling disorder and gaming disorder) that have been classified as “disorders due to addictive behaviors.” While there is growing evidence that addictions to sex, pornography, social network sites, exercise, work, and online shopping may be genuine disorders among a minority of individuals, none of these behaviors is likely to be included in formal psychiatric manuals in the near future until there is more high‐quality data on all research fronts (e.g., epidemiological, neurobiological, psychological, and clinical) (Griffiths, 2022).

The increasing research interest in this phenomenon has led to the development of numerous scales. Laconi et al. (2014) identify 45 measuring instruments (23 languages) and report on their psychometric properties. Among the different tools, literature suggests the Internet Addiction Test (IAT) (Young, 1998), the Generalized Problematic Internet Use Scale (GPIUS) (Caplan, 2002) and its second version (GPIUS‐2) (Caplan, 2010), the Problematic Internet Use Questionnaire (PIUQ) (Demetrovics et al., 2008), and the Compulsive Internet Use Scale (CIUS) (Meerkerk et al., 2009). Specifically, in Spanish populations the CIUS, the GPIUS‐2, the Internet‐Related Experiences Questionnaire (IREQ) (Beranuy et al., 2009), and the PIUS‐a (Rial, Gómez, Sorna, et al., 2015) are the most used instruments. Compared to the GPIUS‐2 (Caplan, 2010; Gámez‐Guadix et al., 2013; Marzo et al., 2022), the CIUS (Meerkerk et al., 2009; Ortuño‐ Sierra et al., 2022) comprised one item less and has been used in long epidemiological surveys conducted in Spain (e.g., Drug Use in Secondary Education Survey [*Encuesta sobre uso de drogas en Enseñanzas secundarias en España, ESUDES*]). In addition, the response format is a 5‐point Likert‐type scale instead a 6‐point Likert‐type scale. CIUS (Meerkerk et al., 2009) is one of the most frequently internationally adapted psychometric instrument developed to assess PIU. It is a well‐validated tool and demonstrated adequate psychometric properties in terms of reliability and validity evidences in various countries and across cultures (Dhir et al., 2016; López‐Fernández et al., 2019; Sarmiento et al., 2021). This scale originally included 14 items related to aspects such as *loss of control* (items 1, 2, 5, and 9), *preoccupation* (items 4, 6, and 7), *withdrawal symptoms* (item 14), *coping* (items 12 and 13), and *conflict* (items 3, 8, 10, and 11) with a 5‐point Likert‐type scale ranging from “never” to “very often.” It was constructed based on criteria of substance abuse dependency and pathological gambling described in the Dianostic and Statistical Manual of Mental Disorder IV (DSM‐IV) along with literature on behavioral addictions (Meerkerk et al., 2009).

Instead of elaborating new instruments, translating and adapting existing validated scales can be especially useful. Laconi et al. (2014) suggested that the number of items for detecting PIU varies between 7 and 91 depending on the instrument. López‐Fernández et al. (2019) tested the psychometric properties of four CIUS versions (i.e., CIUS‐14, CIUS‐9, CIUS‐7, and CIUS‐5) across eight languages (i.e., German, French, English, Finnish, Spanish, Italian, Polish, and Hungarian) to examine their psychometric properties. Findings suggested that the short CIUS‐5 was robust for screening CIU in adults. Besser et al. (2017) developed a short screening instrument by reducing an established 14‐item CIUS to five items (1, 3, 5, 11, and 12). These items showed best performance in the logistic regression analysis. The performance of the abbreviated five‐item version was compared with a second short version, with an additional two items (7 and 9), and the original CIUS using ROC curves and McClish test. Comparison of the AUC and ROC revealed no difference of predictive validity in the three versions of the CIUS. Therefore, the shortest version with five items was defined as the CIUS‐S.

Despite studies analyzing the psychometric adequacy of the CIUS‐S in other languages, there are no studies, to the best of our knowledge, about the psychometric properties of the short form on the CIUS in its Spanish version in representative samples of the adolescent population. The main goal of this paper was to develop the short form of the CIUS (Meerkerk et al., 2009) in its Spanish version and in an adolescent population, specifically, (a) to study the prevalence of PIU among adolescents; (b) to obtain evidence of internal structure of the CIUS‐S; (c) to estimate the reliability of the scores of the CIUS‐S; and (d) to analyze the correlation between the CIUS‐S scores and measures of socioemotional well‐being and mental health indicators.

## 2 METHOD

### 2.1 Participants

Data came from a population of 15,000 students in La Rioja (northern Spain). Stratified random cluster sampling was used, with the classroom as the sampling unit. Students were recruited from 30 schools’ centers and a total of 98 classrooms. The layers were created as a function of the geographical zone and the educational stage. Initially, the sample was composed of 1972 students. However, students with an age higher than 19 years old (*n* = 36) or a high score in the Oviedo Infrequency Scale—Revised (Díez‐Gómez et al., 2020) (more than 2 points) (*n* = 146) were eliminated. The final sample was composed of 1790 students, 961 (53.7%) women, 816 men (45.6%), and 13 gender diversity (0.7%). Age ranged between 14 and 18 years old and the mean age was 15.70 years old (*SD* = 1.26). A total of 89.4% of participants were from Spain, 2.5% Romania, 1.9% Central and South American countries (Bolivia, Argentina, Colombia, and Ecuador), 1.4% Morocco, 0.8% Pakistan, 0.3% Portuguese, and 3.7% from other countries.

### 2.2 Instruments

#### 2.2.1 Compulsive Internet Use Scale

The CIUS (Meerkerk et al., 2009) originally included 14 items on a Likert‐type scale of five options (0 = *never*; 4 = *very often*) to assess PIU. It has showed adequate evidences of factorial, content, and concurrent validity and good evidences of reliability of the scores (Cronbach's *α* between .89 and .90). In addition, other CIUS versions such as CIUS‐9 (Khazaal et al., 2012) or the CIUS‐7 and CIUS‐5 (Besser et al., 2017) have also shown adequate psychometric properties (Dhir et al., 2016; López‐Fernández et al., 2019; Sarmiento et al., 2021).

#### 2.2.2 Adolescent Suicidal Behavior Assessment Scale

The Adolescent Suicidal Behavior Assessment Scale (SENTIA) (Díez‐Gómez et al., 2020) is a self‐report instrument formed by 16 items to screen for suicidal behavior in adolescents. It measures a general suicidal behavior factor and three specific factors (act/planning, communication, and ideation) with a dichotomy format (yes/no). Previous studies have shown that the SENTIA scores have adequate psychometric properties (Díez‐Gómez et al., 2020).

#### 2.2.3 Strengths and Difficulties Questionnaire

The Strengths and Difficulties Questionnaire (SDQ) (Goodman, 1997) has a Likert‐type response format with three options (0 = *not true*, 1 = *somewhat true*, and 2 = *certainly true*). In the present study, the translated and validated Spanish self‐reported version of the SDQ was used (Ortuño‐Sierra et al., 2015). It is composed of 25 items distributed across five subscales (each with five items): emotional problems, behavior problems, peer problems, hyperactivity, and prosocial behavior. Previous studies have shown adequate psychometric properties in Spanish adolescents (Ortuño‐Sierra et al., 2015).

#### 2.2.4 Rosenberg Self‐Esteem Scale

The Rosenberg Self‐Esteem Scale (RSES) (Rosenberg, 2015) is a one‐dimensional scale composed of 10‐item that measures global self‐worth by both positive and negative feelings about the self. All items are answered using a 4‐point Likert‐type scale format ranging from *strongly agree* to *strongly disagree*. In the present study, the Spanish version (Oliva‐Delgado et al., 2014) has been used.

#### 2.2.5 Reynolds Adolescent Depression Scale—Short Form

The Reynolds Adolescent Depression Scale—Short Form (RADS‐SF) (Reynolds, 2004) has 10 items in a Likert‐type scale format with four different options (1 = *almost never*; 4 = *almost always*). It is a self‐reported tool for screening the severity of depressive symptoms (anhedonia, somatic complains, negative evaluation, and dysphoria) in adolescents aged 11–20 years. In the present study, the adapted and validated Spanish version has been used (Ortuño‐Sierra et al., 2017).

#### 2.2.6 Prodromal Questionnaire—Brief

Psychotic‐like symptoms were assessed using the Prodromal Questionnaire—Brief (PQ‐B) (Loewy et al., 2011), which consists of 21 dichotomous items (true/false) and two additional Likert‐type scale questions. Each PQ‐B item is first rated based on whether one has ever experienced a symptom, and then according to how much distress the symptom causes, on a 5‐point scale ranging from *strongly agree* to *strongly disagree*. Previous studies have shown adequate psychometric properties of the instrument (Omega total score = 0.92 and unidimensional factor structure) (Fonseca‐Pedrero et al., 2016).

#### 2.2.7 Maryland Safe and Supportive Schools

A total of 56 items contribute to the Maryland Safe and Supportive Schools (MDS3) (Bradshaw et al., 2014). For the present study, we chose 14 items related to safety, school environment, and student connectedness. All answer choices were rated on a 4‐point Likert‐type scale ranging from *strongly agree* to *strongly disagree*. The adapted and validated Spanish version of the MDS3 was used (Díez‐Gómez et al., 2020).

#### 2.2.8 Oviedo Infrequency Scale‐Revised

This was administered to detect those participants who responded in a random, pseudorandom, or dishonest manner. The Oviedo Infrequency Scale—Revised (INF‐OV‐R) (Díez‐Gómez et al., 2020) is a self‐report instrument composed of 12 items in a 5‐point Likert‐type scale (1 = *completely disagree*; 5 = *completely agree*). In the present study, students with more than two incorrect responses on the INF‐OV‐R scale were eliminated from the sample.

### 2.3 Procedure

The questionnaires were administered collectively by computers, in groups of 10–30 students, during normal school hours. With the aim to standardize the administration process, all the researchers had a protocol that they had to follow before, during, and after conducting the administration of the questionnaires. Participants were informed of the confidentiality of their responses and the voluntary nature of the study. No incentive was provided for their participation. All parents or legal guardian were asked to provide a written informed consent for their child to participate in the study. The present study was approved by the Ethical Committee of Clinical Research of La Rioja.

### 2.4 Data analysis

First, we calculated the descriptive statistics and the percentage distribution of the CIUS‐S’ items. Second, with the aim of gathering evidence about the internal structure of the CIUS‐S, we conducted a Confirmatory Factor Analysis (CFA) attending to the unidimensional model of five items (items 1, 3, 5, 11, and 12) proposed by Besser et al. (2017). The parameters were obtained from the Muthen's quasi‐likelihood estimator. The following goodness‐of‐fit indices were used: Chi square (*χ* ^2^), Confirmatory Factor Index (CFI), Tucker–Lewis Index (TLI), Root Mean Square Error of Approximation (RMSEA), and Standardized Root Mean Square Residual (SRMR). In order to obtain an adequate model fit, Hu and Bentler (1999) suggested that RMSEA values should be under .08 for a good model. Also, CFI and TLI values should be .95 or more, though any value over .90 is considered acceptable. For SRMR, values less than .08 are considered acceptable. Third, we conducted successive multigroup CFAs with the aim to study the Measurement Invariance (MI) by gender and age. We performed multigroup comparisons through structural equation modeling under the measurement models (Byrne, 2008). Considering that the ∆*χ* ^2^ has shown several limitations as it is very sensitive to sample size, Cheung and Rensvold (2002) proposed the increase or decrease in CFI values (∆CFI) to determine if nested models are practically equivalent. We, in addition, calculated latent mean differences across gender and age. To this end, we fixed the latent mean values to zero in the male and in the lower age group. With the aim to stablish comparisons among the studied variables, statistical significance was based on the *z*‐statistic. We considered the reference group the one in which the latent mean was fixed to zero. Fifth, we analyzed the internal consistency of the scores. We used McDonald's Omega as an estimator of the reliability of the scores.

As a final step, we studied the sources of validity evidence of the CIUS‐S with external variables. Thus, we analyzed the correlation between the CIUS‐S scores and measures of –well‐being and mental health by means of Pearson's correlations. SPSS 24.0 (IBM, 2016) and JASP (JASP, 2019) were used for data analyses.

## 3 RESULTS

### 3.1 Descriptive statistics for the CIUS‐S and prevalence rates

Descriptive statistics for the five‐item unidimensional model are depicted in Table 1. In addition, percentage of the different answers’ options for the CIUS‐S is included. For example, 10.1% of adolescents answered “very often” to Item 12 (*How often do you go to the Internet when you are feeling down?*). In addition, the distribution of the scores was studied. With this regard, the CIUS total score was nonnormally distributed, according to the Kolmogorov–Smirnov test = .076 (*p* < .01).

**TABLE 1:** Prevalence rates for the Compulsive Internet Use Scale—Short (CIUS‐S) and descriptive statistics for the different items in the total sample.

Prevalence (%)						Descriptive statistics			
How often…	Never	Rarely	Sometimes	Often	Very often	Mean	*SD*	Skewness	Kurtosis
Item 1. do you find it difficult to stop using the Internet when you are online?	9.6	32.3	34.9	17.8	5.4	1.77	1.02	0.24	–0.45
Item 3. do others (e.g., parents) say you should use the Internet less?	15	26.4	31	18.4	9.2	1.81	1.17	0.17	–0.78
Item 5. are you short of sleep because of the Internet?	24.7	32.2	24.6	12.9	5.6	1.42	1.16	0.50	–0.57
Item 11. do you neglect your daily obligations (school, or family life) because you prefer to go on the Internet?	33.2	35.2	20.7	8.2	2.6	1.12	1.05	0.75	–0.07
Item 12. do you go on the Internet when you are feeling down?	14.5	22.9	29.4	23.1	10.1	1.91	1.20	0.01	–0.90

Abbreviation: *SD*, standard deviation.

### 3.2 Evidence of validity based on internal structure

Table 2 shows the goodness‐of‐fit indices for the dimensional model tested. First, we analyzed the version with five items (one‐dimensional model). This model displayed adequate goodness‐of‐fit indices. Factor loadings for this version were also all statistically significant and over .30. Results are depicted in Table 3.

**TABLE 2:** Goodness‐of‐fit indices for the hypothetical model tested and measurement invariance across gender and age.

Model	*χ* ^2^	*df*	CFI	TLI	RMSEA (IC 90%)	SRMR	AIC	BIC	ΔCFI
5‐items 1 factor	48.275	5	.973	.946	.063 (.052–.088)	.027	25,824.172	25,879.072	
Measurement invariance									
Gender									
Male (*n* = 816)	48.970	5	.975	.954	.068 (.061–.075)	.033	29,862.363	25,083.471	
Female (*n* = 961)	47.872	5	.974	.969	.069 (.062–.075)	.029	35,312.734	25,542.167	
Configural invariance	48.196	10	.976	.952	.066 (.048–.085)	.028	25,551.316	25,660.969	
Metric invariance	54.020	14	.975	.964	.057 (.041–.073)	.034	25,549.140	25,636.863	–.01
Scalar invariance	117.954	18	.937	.93	.079 (.066–.093)	.046	25,625.073	25,745.692	+.01
Age									
14–15 years old (*n* = 883)	48.264	5	.977	.955	.066 (.043–.072)	.032	65,267.465	25,445.632	
16–18 years old (*n* = 907)	47.823.	5	.976	.951	.063 (.045–.080)	.026	33,267.809	25,427.541	
Configural invariance	46.391	10	.977	.954	.064 (.046–.083)	.027	25,620.008	25,729.662	
Metric invariance	49.585	14	.978	.968	.053 (.038–.070)	.030	25,615.201	25,702.924	–.01
Scalar invariance	51.787	18	.966	.962	.059 (.045–.073)	.032	25,650.604	25,771.223	+.01

Abbreviations: AIC, Akaike information criterion; BIC, Bayesian information criterion; CFI, Comparative Fit Index; *df*, degrees of freedom; IC, interval confidence; RMSEA, Root Mean Square Error of Approximation; SRMR, Standardized Root Mean Square Residual; TLI, Tucker–Lewis Index; ΔCFI, change in Comparative Fix Index.

**TABLE 3:** Factor loadings for the CIUS‐S final one‐dimensional model with five correlated errors.

			CI 95%	
Item	Factor loadings	*SE*	Lower	Upper
1	0.666	0.023	0.621	0.711
3	0.682	0.027	0.629	0.735
5	0.649	0.027	0.596	0.701
11	0.659	0.024	0.613	0.706
12	0.635	0.028	0.580	0.690

Abbreviations: CI, confidence interval; *SE*, standard error.

### 3.3 Measurement invariance of the CIUS‐S’ scores by gender and age

We studied the MI across gender and age in this model. With the aim to study MI by gender, only male and female were considered, due to the limited amount of people not identifying a gender (*n* = 13). In order to study the MI by age, we divided the sample into two different groups—first group (adolescents from 14 to 16 years old) and second group (adolescents from 17 to 18 years old)—attending to the Spanish educational system (compulsory/post compulsory). As can be seen in Table 2, the ΔCFI lower than .01 permits confirming strong MI both by gender and age.

### 3.4 Analysis of latent mean scores

The comparison across groups in latent means revealed statistically significant differences in the CIUS Total Score. Therefore, the comparison across groups in latent means indicated that, on average, men scored 0.183 units under women in the CIUS Total Score (*p* < .01). With regard to the age, comparison across groups in latent means revealed no statistically significant differences between the younger and the older group.

### 3.5 Study of the reliability of the CIUS‐S’ scores

With the aim to study the internal consistency of the CIUS‐S scores, we calculated the McDonald's Omega coefficient. The total score displayed a coefficient of 0.73. All the discrimination indices were over .30.

### 3.6 Relation of the CIUS‐S’ with well–being and mental health variables

Finally, we analyzed the correlation between the CIUS‐S’ scores and variables related to socioemotional adjustment. Results are depicted in Table 4. CIUS‐S’ scores were positively associated with suicidal behavior, depressive symptoms, emotional and behavioral problems, and psychotic like experiences. Moreover, CIUS‐S’ scores were negatively correlated with self‐esteem, prosocial behavior, and the feeling of belonging. In addition, the CIUS‐S’ scores were very strongly associated with the CIUS original form (*r* = .927).

**TABLE 4:** Pearson's correlation matrix between the CIUS‐S’ scores and different indicators of well‐being and mental health.

Well‐being and mental health variables	CIUS‐S
Suicidal behaviors	.273^*^
Psychotic like experiences	.162^*^
Depressive symptoms	.399^*^
Emotional problems	.322^*^
Behavior problems	.237^*^
Peer problems	.147^*^
Hyperactivity	.284^*^
Prosocial behaviors	–.148^*^
Self‐esteem	–.347^*^
Feeling of belonging	–.183^*^
CIUS	.927^*^

*p < .01.

## 4 DISCUSSION

Today, Internet access and use for children and adolescents in European countries is almost ubiquitous (Anderson et al., 2017; Smahel et al., 2020). Time spent online has increased during and since the world pandemic COVID‐19, not only for adults but also for adolescents. Thus, PIU is receiving an increased amount of attention, and more research is devoted to understand and screen for a phenomenon that has been associated with different problems including, among others, sleeping and eating disorders, social skills deficits, sedentary lifestyles, family conflicts, or poor school performance (Golpe‐Ferreiro et al., 2017; Rial et al., 2015; Vila et al., 2018). Bearing that in mind, screening at early stages seems increasingly relevant and assessment tools seem highly necessary. Therefore, the main goal of the present study was to analyze the psychometric adequacy of a short form of the CIUS in its Spanish version.

Results with regard to the internal structure suggest that the five‐item version unidimensional model had adequate goodness‐of‐fit indices. Previous results with shorter forms are, however, contradictory. For example, Lopez‐Fernandez et al. (2019) revealed that the five‐item short version of the CIUS worked well in different countries. In addition, Dhir et al. (2015) found that an eight‐item version had adequate psychometric properties. The study of Besser et al. (2017) indicated that both the five‐ and the seven‐item versions were adequate. Nonetheless, they retained the five‐item version as better discrimination indices were found.

Another critical aspect of a measurement instrument is the fact that its dimensional structure could be replicated by attending to the different variables of the object of study (e.g., gender, age, culture, etc.). With this regard, we studied the MI of the five‐item version by gender and age. The results indicated that partial MI was tenable for both gender and age. Previous studies, similarly to our results, revealed MI of the CIUS by gender, as well as Internet use (Meerkerk et al., 2009). However, few studies have analyzed the MI of the instrument in it shorter form. For instance, Gmel et al. (2019) revealed that the short form of eight items was invariant across gender, age, and region. It is worth noting that the study was conducted with a population of young adult participants, so results may not apply to adolescent populations. Studying MI isalso relevant as it contributes essential evidence of construct validity for the shorter form of the CIUS scores in adolescents. We also studied the comparison in the latent means across gender and age. Results found indicated that men scored statistically significantly lower than women overall. This is consistent with previous studies indicating higher prevalence of PIU among women (Andrade et al., 2021; Gómez et al., 2017; Rial et al., 2015; Rial et al., 2018). For instance, in Spain, recent research indicated a higher percentage among women (36.1%) compared to men (29.8%) (Andrade et al., 2021). These results are relevant in order to understand the manifestation of CIUS during adolescence. This stage of life is a critical developmental period with different biopsychological changes that have, potentially, a different impact with regard to the gender and the age of the person (Liu et al., 2022).

With regard to validity evidences based on relations with other variables, the CIUS‐S revealed positive and statistically significant associations with variables related to mental health such as emotional difficulties, behavioral problems, psychotic experiences, depressive symptoms, and suicidal behaviors. The association was negative and statistically significant with prosocial behavior and feeling of belonging. In addition, the CIUS‐S and the CIUS long form were also correlated. Other studies have also revealed correlations between PIU and mental health problems like depressive symptoms and insomnia (Jain et al., 2020). In addition, problematic internet use is correlated with mood symptoms (Gao et al., 2020), as well as substance abuse and suicidal behavior (Serrano et al., 2017). Considering the prevalence of PIU behaviors during adolescence and the associations between PIU and different mental health problems, it may be important to screening both in school and clinical settings.

The present study has some limitations. First, it is based on self‐reported measures, and there are well‐known problems related to this kind of measures. Therefore, experimental data or studies including parents, teachers, or relatives could add valuable information to this area of study. In addition, we conducted a cross‐sectional study, which limits establishing cause–effect associations. Thus, longitudinal studies analyzing PIU are still needed.

Notwithstanding the mentioned limitations, the present work provides valuable information for the screening of PIU in adolescent populations. This is, to the best of our knowledge, the first study providing evidence of the psychometric adequacy of a short form of the CIUS in its Spanish version and in adolescents’ populations. To sum up, the CIUS‐S is a brief and easy‐to‐use screening instrument that allows study of this increasing phenomenon.

## CONFLICT OF INTEREST STATEMENT

The authors declare no conflicts of interest.

### PEER REVIEW

The peer review history for this article is available at https://publons.com/publon/10.1002/brb3.3133.

## Data Availability

The data presented in this study are available on request from the corresponding author.
